# Circulating extracellular vesicle-carried PTP1B and PP2A phosphatases as regulators of insulin resistance

**DOI:** 10.1007/s00125-024-06288-0

**Published:** 2024-10-18

**Authors:** Sakina Ali, Xavier Vidal-Gómez, Megan Piquet, Luisa Vergori, Gilles Simard, Séverine Dubois, Pierre-Henri Ducluzeau, Pascal Pomiès, Sarah Kamli-Salino, Mirela Delibégovic, Samir Henni, Frédéric Gagnadoux, Ramaroson Andriantsitohaina, M. Carmen Martínez, on behalf of the Metabol Study Group

**Affiliations:** 1 SOPAM, U1063, Inserm, UNIV Angers, SFR ICAT, Angers, France; 2 University of Montpellier, PhyMedExp, Inserm, CNRS UMR, Montpellier, France; 3 Centre Hospitalo-Universitaire d’Angers (CHU), Angers, France; 4 Service de Médecine Interne, Unité d’Endocrinologie Diabétologie et Nutrition, Centre Hospitalier Universitaire et Faculté de Médecine, Université de Tours, Tours, France; 5 Aberdeen Cardiovascular and Diabetes Centre, Institute of Medical Sciences, University of Aberdeen, Aberdeen, UK; 6 Département de Pneumologie et Médecine du Sommeil, CHU d’Angers, Angers, France

**Keywords:** Adipose tissue, Extracellular vesicles, Insulin resistance, Liver, Phosphatases

## Abstract

**Aims/hypothesis:**

Metabolic disorders associated with abdominal obesity, dyslipidaemia, arterial hypertension and hyperglycaemia are risk factors for the development of insulin resistance. Extracellular vesicles (EVs) may play an important role in the regulation of metabolic signalling pathways in insulin resistance and associated complications.

**Methods:**

Circulating large EVs (lEVs) and small EVs (sEVs) from individuals with (IR group) and without insulin resistance (n-IR group) were isolated and characterised. lEVs and sEVs were administered by i.v. injection to mice and systemic, adipose tissue and liver insulin signalling were analysed. The role of phosphatases was analysed in target tissues and cells.

**Results:**

Injection of lEVs and sEVs from IR participants impaired systemic, adipose tissue and liver insulin signalling in mice, while EVs from n-IR participants had no effect. Moreover, lEVs and sEVs from IR participants brought about a twofold increase in adipocyte size and adipogenic gene expression. EVs from IR participants expressed two types of phosphatases, phosphotyrosine 1 phosphatase (PTP1B) and protein phosphatase 2 (PP2A), IR lEVs being enriched with the active form of PTP1B while IR sEVs mainly carried active PP2A. Blockade of PTP1B activity in IR lEVs fully restored IRS1 and Akt phosphorylation in adipocytes and blunted insulin-induced Akt phosphorylation by inhibition of the macrophage secretome in hepatocytes. Conversely, blockade of PP2A activity in IR sEVs completely prevented insulin resistance in adipocytes and hepatocytes.

**Conclusions/interpretation:**

These data demonstrate that inhibition of phosphatases carried by EVs from IR participants rescues insulin signalling in adipocytes and hepatocytes and point towards PTP1B and PP2A carried by IR EVs as being novel potential therapeutic targets against insulin resistance in adipose tissue and liver and the development of obesity.

**Graphical Abstract:**

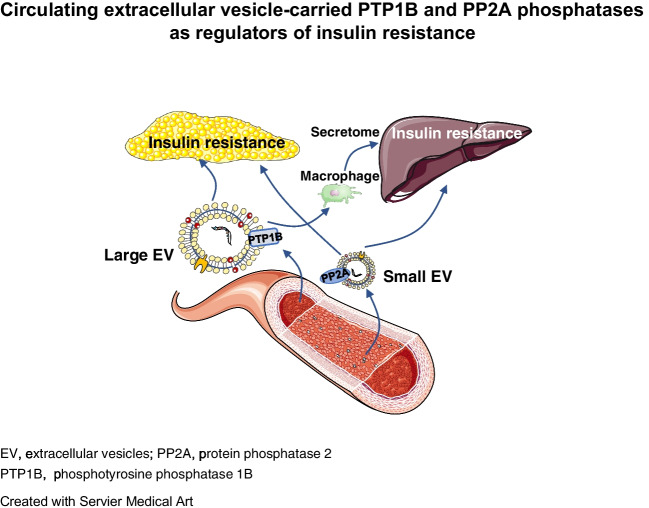

**Supplementary Information:**

The online version of this article (10.1007/s00125-024-06288-0) contains peer-reviewed but unedited supplementary material.


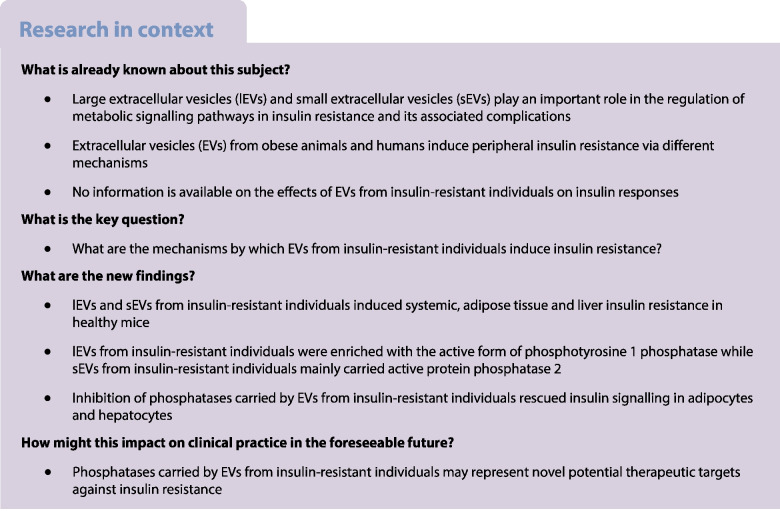


## Introduction

Because of their ability to transfer their contents into recipient cells, extracellular vesicles (EVs), including large EVs (lEVs) and small EVs (sEVs), mediate intercellular crosstalk under pathophysiological conditions [1–5]. Several studies have shown an alteration in the number of EVs or their cargo in metabolic diseases, including type 2 diabetes and obesity, suggesting that EVs may play an important role in the regulation of metabolic signalling pathways in diabetes and associated complications (for review see [6–8]). It has been shown that sEVs derived from adipose tissue of obese mice induce insulin resistance in healthy mice, through mechanisms involving TNF-α and Toll like receptor-4 (TLR4) [9]. In addition, macrophages from adipose tissue of obese mice release microRNA-enriched sEVs, which trigger glucose and insulin intolerance in healthy mice as well as insulin resistance in liver, muscle and adipose tissue [5]. Moreover, sEVs from plasma of obese humans induce insulin resistance in hepatocytes and adipocytes by decreasing Akt activation and glucose transport [10, 11]. However, the effects of lEVs on insulin responses have not been reported.

Here, we investigated the ability of lEVs and sEVs from insulin-resistant individuals to induce systemic, adipose tissue and liver insulin resistance in healthy mice. The mechanisms involved in EV-induced insulin resistance were also analysed in cultured cells. Independently of their miRNA content, EVs carry key proteins regulating the insulin pathway. Among these proteins, we have focused the present study on the role of EV-carried phosphatases, such as phosphotyrosine phosphatase 1B (PTP1B) and protein phosphatase 2 (PP2A), as actors in the negative modulation of insulin sensitivity [12–14].

## Methods

For detailed methods, please refer to the electronic supplementary material (ESM).

### Participants

This study was approved by the Ethics Committee of the University Hospital Centre of Angers (France) (NUMEVOX cohort, ClinicalTrials.gov registration no. NCT00997165). Patients at the Department of Endocrinology and Nutrition of the University Hospital of Angers were recruited as participants. After giving informed consent, cohort participants were characterised according to the HOMA-IR index. A total of 67 participants were identified as being insulin resistant (IR group; HOMA-IR ≥1.7); 38 participants were identified as non-insulin-resistant (n-IR group; HOMA-IR <1.7). Baseline characteristics and clinical data for participants in the n-IR and IR groups are summarised in ESM Table 1. Race and ethnicity data were not available in this study.

### EV isolation

Peripheral blood from participants was collected in EDTA tubes to isolate circulating EVs [1, 15].

### Electronic transmission microscopy

EV preparations were first fixed overnight at 4°C with 2.5% glutaraldehyde in 0.1 mol/l PBS, as described in ESM Methods.

### Characterisation of EV-associated proteins

Purified EVs were separated on 4–15% Criterion TGX precast gels and transferred onto nitrocellulose membranes. To characterise EVs, blots were probed as described in ESM Methods. Antibodies are shown in ESM Table 2.

### Flow cytometry and nanoparticle tracking analysis

Counting and phenotyping of lEVs were performed by flow cytometry according to the expression of membrane-specific antigens. The size and concentration of sEVs were assessed using the NanoSight NS300.

### Animals

All animal studies were performed using approved institutional protocols (nAPAFiS no. 22561 and no. 47728-2024022218479296 v6) (see ESM Methods).

### GTT and ITT

Fasting blood glucose levels were measured after 12 h of fasting, then mice received an i.p. injection of 2 g/kg glucose. Blood glucose levels were measured until 120 min after glucose injection. For the ITT, mice were fasted for 6 h before receiving an i.p. injection of 0.5 IU/kg insulin. Blood glucose levels were measured every 15 min for 1 h.

### In vivo insulin-induced Akt phosphorylation assay

To evaluate insulin action in tissues, insulin-induced Akt phosphorylation was measured in visceral adipose tissue, and liver as described by Ying et al [5]. Mice were fasted for 8 h, then anaesthetised and portions of these tissues were harvested to measure basal levels of Akt phosphorylation. Then, mice were injected with insulin into the vena cava and portions of the liver and visceral adipose tissues were collected at 3 min and 7 min, respectively, with these time points predetermined to obtain maximal Akt phosphorylation.

### Histological analysis

Tissue sections were stained with H&E. Quantification of adipocyte size was performed using ImageJ software.

### Cell culture

HepG2 and mature 3T3L1 cells were serum-deprived in DMEM low glucose (5.5 mmol/l glucose) supplemented with 0.5% BSA. Then, cells were treated for 24 h with a circulating concentration of lEVs. For sEV treatment, cells were treated with 10 µg/ml sEVs. After 24 h of treatment, cells were stimulated with 100 nmol/l insulin for 5 min. To block the effect of phosphotyrosine phosphatase 1B (PTP1B) carried by lEVs, 50 μmol/l BML-267 or 10 µmol/l MSI-1436 were added to isolated lEVs for 1 h at 4°C before pelleting the lEVs by centrifugation at 21,000 *g* for 45 min. To inhibit the action of protein phosphatase 2 (PP2A) carried by sEVs, 0.5 nmol/l okadaic acid was added to isolated sEVs for 1 h at 4°C. sEVs were pelleted with ExoQuick followed by centrifugation at 1500 *g* for 30 min. Then, cells were incubated with either BML-267-, MSI-1436-treated lEVs or okadaic acid-treated sEVs for 24 h followed by insulin stimulation with 100 nmol/l insulin for 5 min.

### Glycogen assay

The glycogen assay was measured using a specific kit (see ESM Methods).

### Western blotting

After EV treatment, proteins were separated from lysed cells or tissues. Blots were probed with antibodies (see ESM Table 1).

### Phosphatase activity assay

EVs were resuspended in lysis buffer containing 20 mmol/l imidazole, 2 mmol/l EGTA, 1 mmol/l phenylmethylsulfonyl fluoride and protease inhibitors. Phosphatase activity was measured using a specific pNPP hydrolysis kit for PTP1B and PP2A.

### Human macrophage culture and multiplex immunoassays for macrophage secretome

Macrophages were treated with lEVs for 24 h in the absence or presence of MSI-1436 (10 µmol/l). Supernatant fractions were used for analysis using multiplex assays.

### Quantification of neutral lipids

Cells were stained with Oil-Red-O as described in ESM Methods. Adipocyte and lipid droplet size were quantified by Image J software.

### Quantitative real-time PCR analysis

RNA was extracted from mouse adipose tissue and quantitative real-time PCR (qPCR) was performed as described in ESM Methods. The primers are presented in ESM Table 1.

### Statistical analysis

Comparisons were made between all conditions. Statistical analysis was performed using ANOVA, followed by Kruskal–Wallis test for multiple comparisons of more than two groups; for comparisons between two groups, data were analysed using the Mann–Whitney *U* test. When measurements were repeated in the same group of animals, a two-way ANOVA followed by Tukey’s multiple comparisons test was performed. No experiment-wide multiple test correction was applied. Values shown in the text and figures represent the mean ± SEM or median (IQR). Each data point represents EV isolation from one participant. A *p* value ≤0.05 was considered statistically significant. Statistical analysis was performed using GraphPad Prism 8.0 (GraphPad Software, San Diego, CA, USA).

## Results

### lEV and sEV characterisation

Electron microscopy (ESM Fig. 1a, b) and nanoparticle tracking (ESM Fig. 1c) analyses revealed that lEVs and sEVs from IR participants had a mean diameter of ~450 and ~100 nm, respectively. sEVs from IR participants were smaller in size than those from n-IR participants (ESM Fig. 1c). lEVs but not sEVs expressed the cytoskeletal protein β-actin, as previously described, whereas sEVs were enriched in CD9, CD63 and CD81 when compared with lEVs (ESM Fig. 1d). Neither lEVs nor sEVs expressed Grp94, GM130 or ApoA1, showing the purity of isolated EVs. Circulating lEV levels in IR participants, including those from platelets (CD41^+^) or endothelial cells (CD146^+^), and sEV levels in IR participants were significantly increased when compared with n-IR participants (ESM Table 1, ESM Fig. 1e) [1, 16].

### lEVs and sEVs from IR participants promote insulin resistance in mice

**Fig. 1:**
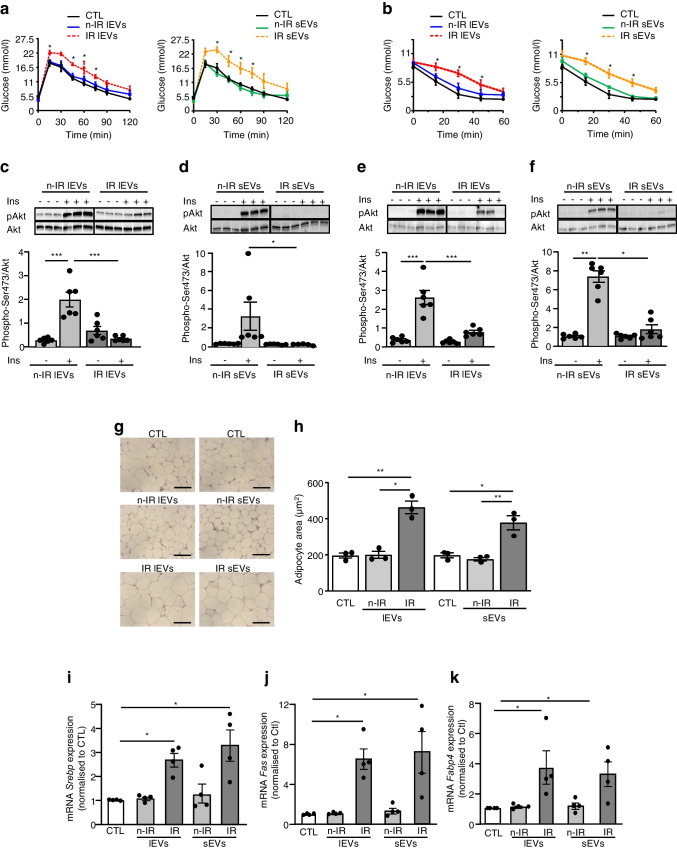
lEVs and sEVs from IR participants cause insulin resistance and increase adipocyte size in mice. (**a**–**f**) Mice were injected every 7 days/2 weeks with circulating levels of lEVs or 30 µg sEVs from n-IR or IR participants, then a GTT (**a**, *n*=7 mice/group) and ITT (**b**, *n*=7 mice/group) were performed. *p* values were determined by repeated-measures two-way ANOVA followed by a Tukey’s multiple comparisons test. *p<0.05 vs control. In vivo insulin-induced Akt phosphorylation at Ser473 was measured in adipose tissue (**c**, **d**, *n*=6 mice/group) and liver (**e**, **f**, *n*=6 mice/group). The black line on the immunoblots indicates when samples were loaded on the same gel but not side by side. (**g**, **h**) Adipocyte size in visceral adipose tissue of mice. Each point represents the mean of two replicated independent tissue sections, each containing *n*=3 samples. Scale bar, 100 µm. (**i**–**k**) Expression of adipogenic genes in visceral adipose tissue (*n*=4 mice/group). Data are shown as mean ± SEM. ANOVA was carried out followed by the Kruskal–Wallis test. *p<0.05, **p<0.01, ***p<0.001. CTL, control

Neither lEVs nor sEVs from n-IR participants significantly modified body weight when injected into mice (ESM Fig. 2). Injection of lEVs from IR participants induced a non-significant increase (*p*=0.8126) of body weight and adipose tissue mass, whereas injection of sEVs from IR participants increased body weight and adipose tissue mass in mice after 14 days of treatment (ESM Fig. 2). There were no differences in fasting blood glucose levels between the groups of mice (Fig. 1a). lEVs and sEVs from IR participants, but not those from n-IR participants, impaired glucose tolerance and insulin sensitivity in mice (Fig. 1a, b). The ability of EVs to modify in vivo insulin-induced Akt phosphorylation in visceral adipose tissue and in liver was analysed after i.v. injection of insulin into the vena cava [5]. IR lEVs (Fig. 1c) and IR sEVs (Fig. 1d), but not EVs from n-IR participants, abrogated the ability of insulin to induce Akt phosphorylation in visceral adipose tissue. Similarly, insulin-induced Akt phosphorylation in mouse liver was significantly decreased by treatment with either lEVs or sEVs from IR participants but not with EVs from n-IR participants (Fig. 1e, f).

### lEVs and sEVs from IR participants increase adipocyte size in mice

A significant increase in adipocyte size in mice treated with lEVs and sEVs from IR participants compared with EVs from n-IR participants was observed (Fig. 1g, h). In contrast, neither lEVs nor sEVs from n-IR or IR participants modified hepatocyte morphology in mouse liver (ESM Fig. 3a), suggesting a specific effect of IR EVs on adipose tissue lipogenesis. Indeed, both lEVs and sEVs from IR participants induced an increase in *Srebp*, *Fas* and *Fabp4* mRNA levels, which are implicated in the regulation of adipogenic gene expression and in lipid synthesis in adipose tissue (Fig. 1i–k). In contrast, injection of EVs from n-IR participants had no effect on the expression of these genes.

### High levels of PTP1B carried by lEVs from IR participants account for insulin resistance in adipocytes

**Fig. 2:**
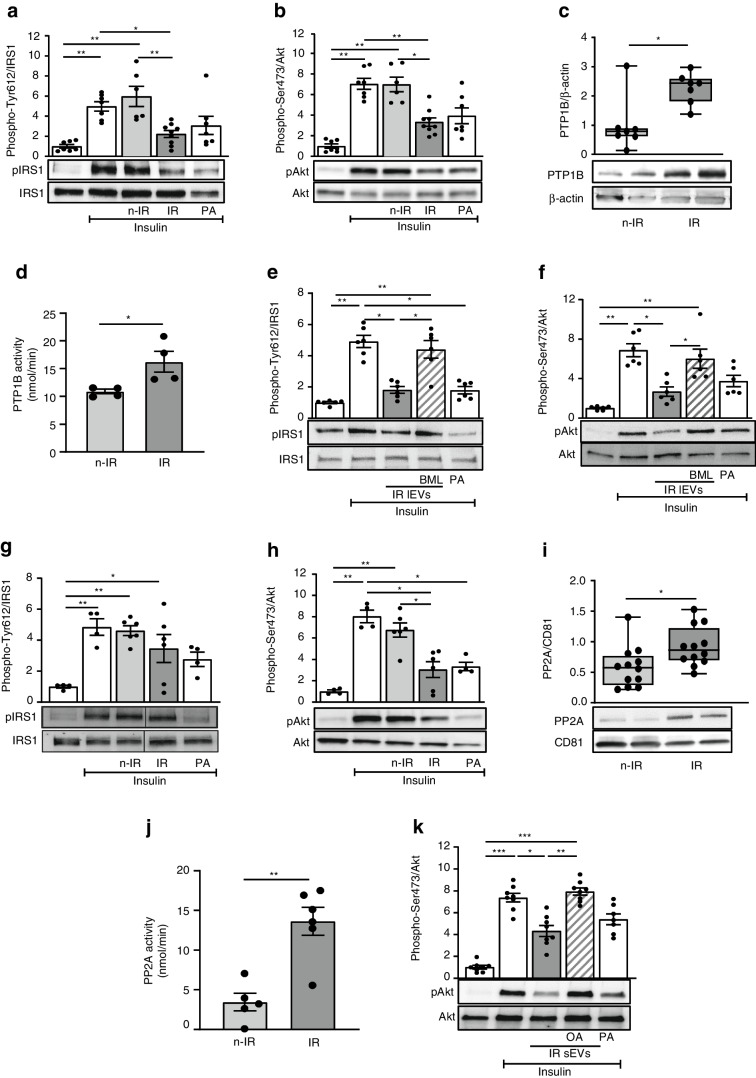
PTP1B and PP2A carried from EVs from IR participants decrease insulin sensitivity in 3T3L1 adipocytes. 3T3L1 adipocytes were untreated or incubated for 24 h with 300 µmol/l palmitic acid or lEVs (**a**–**f**) or sEVs (**g**–**k**) from n-IR or IR participants followed by 100 nmol/l insulin stimulation. (**a**, **b**) Representative immunoblot and quantification of phospho-IRS1 Tyr612 (**a**) and phospho-Akt Ser473 (**b**). *n*=6–9 independent experiments. (**c**, **d**) PTP1B expression (**c**) and activity (**d**) in lEVs from n-IR or IR participants. *n*=4–7 samples/group. (**e**, **f**) Representative immunoblot and quantification of phospho-IRS1 Tyr612 (**e**) and phospho-Akt Ser473 (**f**) in 3T3L1 adipocytes treated with IR lEVs pre-treated with the PTP1B inhibitor BML-267 (50 µmol/l). *n*=6 independent experiments. (**g**, **h**) Immunoblot and quantification of phospho-IRS1 (**g**) and phospho-Akt (**h**) from 3T3L1 adipocytes treated with sEVs from n-IR or IR participants followed by 100 nmol/l insulin stimulation. *n*=4–6 independent experiments. (**i**, **j**) PP2A expression (**i**) and activity (**j**) in sEVs from n-IR or IR participants. *n*=5–12 samples/group. (**k**) Representative immunoblot and quantification of phospho-Akt in 3T3L1 adipocytes treated with IR sEVs pre-incubated with 0.5 nmol/l okadaic acid. *n*=7–8 independent experiments. Data are shown as mean ± SEM, except for (**c**) and (**i**) where the box and whiskers graphs show data distribution (top and bottom quartiles in boxes and minimum and maximum values with lines) and medians. ANOVA was carried out followed by the Kruskal–Wallis test, except for (**d**) and (**j**) in which the Mann–Whitney *U* test was carried out. *p<0.05; **p<0.01; ***p<0.001. BML, BML-267; OA, okadaic acid; PA, palmitic acid

To determine the molecular mechanisms involved in the decreased insulin activation induced by EVs, we specifically analysed the effects of EVs in insulin-target cells. For this, 3T3L1 adipocytes were treated with EVs from n-IR or IR participants or with palmitic acid. Palmitic acid, as a positive control, decreased insulin-induced phospho-IRS1 and phospho-Akt (Fig. 2a, b). Interestingly, lEVs from IR but not n-IR participants significantly decreased insulin-induced IRS1 and Akt phosphorylation in 3T3L1 adipocytes (Fig. 2a, b). We next tested whether phosphatases carried by IR lEVs might be involved in the decrease of insulin-induced IRS1 and Akt phosphorylation. PTP1B plays an important physiological role in the negative modulation of insulin sensitivity by dephosphorylating IRS1 and increases in PTP1B activity have been reported to be involved in the pathogenesis of insulin resistance and diabetes [13]. Here, IR lEVs were enriched in PTP1B and displayed an increase of PTP1B activity when compared with lEVs from n-IR participants (Fig. 2c, d and ESM Fig. 4a). As shown in Fig. 2e, f, inhibition of PTP1B activity with BML-267 [17] on IR lEVs rescued insulin signalling in 3T3L1 adipocytes by preventing IRS1 dephosphorylation, thereby increasing Akt phosphorylation.

### IR sEVs decrease insulin signalling in adipocytes by a PP2A-dependent mechanism

Insulin-induced phosphorylation of IRS1 was not modified by either n-IR or IR sEVs, in contrast to lEVs (Fig. 2g). However, IR sEVs decreased insulin-induced Akt phosphorylation (Fig. 2h); this effect was not prevented when IR sEVs were pre-treated with the PTP1B inhibitor BML-267 (ESM Fig. 4b), suggesting that phosphatases other than PTP1B may be involved in the dephosphorylation of Akt induced by IR sEVs.

We focused on protein phosphatase 1 (PP1) and PP2A, two phosphatases associated with serine dephosphorylation of Akt in metabolic disorders [14, 18]. sEVs from IR participants were enriched in PP2A but not in PP1 (Fig. 2i and ESM Fig. 4a), compared with n-IR sEVs. Similarly, PP2A activity was increased in IR sEVs compared with sEVs from n-IR participants (Fig. 2j). To assess the contribution of PP2A carried by sEVs to insulin resistance, IR sEVs were incubated with okadaic acid, which inhibits PP2A activity, prior to treatment of 3T3L1 adipocytes. Inhibition of PP2A conveyed by IR sEVs prevented Akt dephosphorylation in 3T3L1 adipocytes (Fig. 2k).

### lEVs and sEVs from IR participants increase lipid accumulation in 3T3L1 adipocytes

**Fig. 3:**
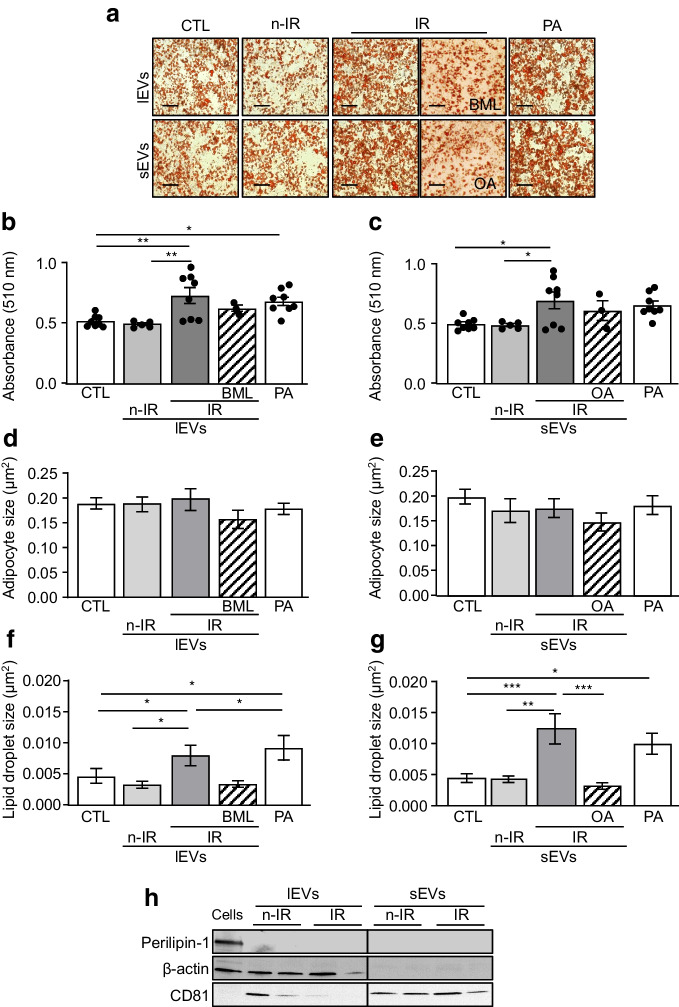
lEVs and sEVs from IR participants increase lipid accumulation in 3T3L1 adipocytes. (**a**–**g**) Lipid content (**a**), quantification by Oil-Red-O staining (**b**, **c**), adipocyte size (**d**, **e**) and lipid droplet size (**f**, **g**) in 3T3L1 adipocytes treated with 300 µmol/l PA or lEVs (**b**, **d**, **f**) or sEVs (**c**, **e**, **g**) from n-IR or IR participants. IR lEVs were pre-treated with the PTP1B inhibitor BML-267 (50 µmol/l) and sEVs were pre-treated with 0.5 nmol/l okadaic acid. *n*=3–8 independent experiments. Scale bar, 50 µm. (**h**) Representative immunoblot of perilipin-1 content in adipocyte lysate and lEVs and sEVs from n-IR or IR participants. Data are shown as mean ± SEM. ANOVA was carried out followed by the Kruskal–Wallis test. *p<0.05, **p<0.01, ***p<0.001. BML, BML-267; CTL, control; OA, okadaic acid; PA, palmitic acid

Both lEVs and sEVs from IR participants significantly increased the number of adipocytes and lipid droplet size in 3T3L1 adipocytes, in the same way as palmitic acid, compared with control cells or cells incubated with n-IR EVs (Fig. 3a–g). Inhibition of PTP1B or PP2A induced a non-significant decrease of Oil-Red-O staining in 3T3L1 adipocytes and a significant decrease in lipid droplet size. To evaluate whether the effects of IR EVs on adipocytes were due to the direct transfer of their lipid content into 3T3L1 adipocytes, we analysed the expression of perilipin-1, a specific protein of lipid droplet complexes. lEVs and sEVs from n-IR and IR participants did not express perilipin-1, as compared with adipocyte lysates, suggesting the absence of lipid droplets in EVs (Fig. 3h). Together, these results suggest that both lEVs and sEVs from IR participants profoundly affect the adipocyte lipid droplets indicating a lipid droplet expansion and lipid accumulation in adipocytes.

### IR lEVs decrease insulin signalling in hepatocytes via the secretion of IL-6 and IL-18 by macrophages

**Fig. 4:**
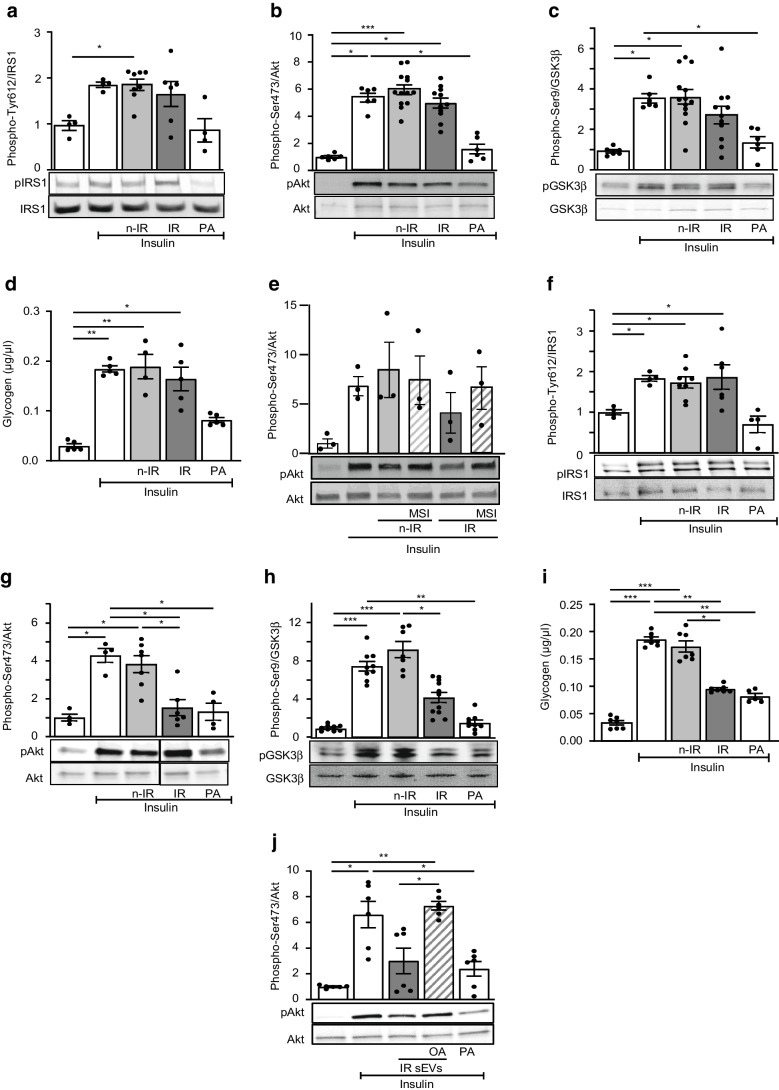
Effects of lEVs and sEVs from IR participants in HepG2 cells. HepG2 cells were untreated or treated for 24 h with 300 µmol/l palmitic acid or lEVs (**a**–**e**) or sEVs (**f**–**j**) from n-IR or IR participants followed by 100 nmol/l insulin stimulation. (**a**–**c**) Representative immunoblot and quantification of phosphorylation of IRS1 Tyr612 (**a**), Akt Ser473 (**b**) and GSK3β Ser9 (**c**). *n*=4–13 independent experiments. (**d**) Glycogen content in cells after lEVs or palmitic acid treatment. *n*=4–5 independent experiments. (**e**) Immunoblot and quantification of phosphorylation of Akt Ser473 in HepG2 cells treated with the conditioned medium derived from human macrophages treated with IR lEVs in the absence or presence of the PTP1B inhibitor MSI-1436 (10 µmol/l). *n*=3 independent experiments. (**f**–**h**) Representative immunoblot and quantification of phosphorylation of IRS1 Tyr612 (**f**), Akt Ser473 (**g**) and GSK3β Ser9 (**h**). *n*=4–11 independent experiments. (**i**) Glycogen content in cells after sEV or palmitic acid treatment. *n*=5–7 independent experiments. (**j**) Representative immunoblot and quantification of phospho-Akt Ser473 in HepG2 cells treated with IR sEVs pre-incubated with 0.5 nmol/l okadaic acid. *n*=6 independent experiments. The black line on the immunoblots indicates when samples were loaded on the same gel but not side by side. Data are shown as mean ± SEM. ANOVA was carried out followed by the Kruskal–Wallis test. *p<0.05, **p<0.01, ***p<0.001. MSI, MSI-1436; OA, okadaic acid; PA, palmitic acid

In HepG2 cells, palmitic acid decreased IRS1, Akt and glycogen synthase kinase-3β (GSK3β) phosphorylation and reduced glycogen synthesis induced by insulin (Fig. 4a–d). Surprisingly, lEVs did not modify insulin-induced IRS1, Akt and GSK3β phosphorylation (Fig. 4a–c) or glycogen synthesis in HepG2 cells (Fig. 4d). Because IR lEVs inhibit insulin signalling in vivo in mouse liver but not in cultured hepatocytes, we analysed whether proinflammatory cytokines released by lEV-treated macrophages account for the insulin resistance in liver. Accordingly, we investigated the contribution of the secretome of human macrophages treated with EVs from n-IR and IR participants to insulin resistance in HepG2 cells. IR lEVs induced an increase in IL-18 and IL-6 secretion from human primary macrophages. IL-18 secretion was significantly inhibited by the PTP1B inhibitor MSI-1436, whereas IL-6 secretion was reduced by ~14% (ESM Fig. 3b). To assess the contribution of these cytokines to insulin resistance induced by lEVs in the liver, HepG2 cells were treated for 24 h with conditioned media from macrophages treated with lEVs in the presence or absence of MSI-1436 (Fig. 4e). Conditioned media from macrophages treated with IR lEVs induced a non-significant decrease (~34%) of Akt phosphorylation in HepG2 cells. Moreover, this effect was attenuated when HepG2 cells were incubated with conditioned media from macrophages treated with IR lEVs and MSI-1436 (Fig. 4e).

### IR sEVs decrease insulin signalling in hepatocytes in an IRS1-independent manner by a PP2A inhibitor-sensitive pathway

sEVs from IR participants did not modify IRS1 phosphorylation but induced a significant decrease in phospho-Akt and phospho-GSK3β in response to insulin in hepatocytes (Fig. 4f–h). HepG2 cells lost their ability to correctly synthesise glycogen under insulin stimulation after treatment with sEVs from IR participants compared with those from n-IR participants (Fig. 4i). These results demonstrate that sEVs from IR participants induce insulin resistance in hepatocytes in an IRS1-independent manner. The decrease in phospho-Akt induced by IR sEVs was not prevented in the presence of the PTP1B inhibitor (ESM Fig. 4c), but blockade of PP2A transported by IR sEVs prevented the dephosphorylation of Akt in HepG2 (Fig. 4j).

Furthermore, neither lEVs nor sEVs from n-IR and IR participants had any effect on lipid synthesis in hepatocytes, when compared with palmitic acid, which significantly increased lipid accumulation in these cells (ESM Fig. 3c, d).

## Discussion

The present study demonstrates that phosphatases carried by EVs from IR individuals induce systemic insulin resistance in mice by reducing the activation of insulin signalling in target tissues such as adipose tissue and liver. Interestingly, EVs induced an increase in body weight due to their ability to increase subcutaneous and visceral but not epididymal adipose tissue; this effect was more pronounced for sEVs compared with lEVs. Furthermore, for both types of EVs the increase in body weight was associated with an increase in adipocyte size and expression of pro-adipogenic factors. These results confirm that increased adipose tissue mass is largely responsible for the observed weight gain. The mechanisms accounting for the effect of EVs on body weight remain unknown. However, several hypotheses can be advanced: (1) since EV treatment induced an increase in adipocyte size and the expression of adipogenic factors, it is possible that the direct effect of EVs on adipocyte metabolism induces lipogenesis and adipogenesis that participate in the weight gain; (2) phosphatases carried by EVs can affect adipogenic differentiation of adipose stem progenitor cells, which correlates with adipose tissue hypertrophy and insulin resistance [19]; (3) an effect on the central regulation of EVs on energy metabolism cannot be ruled out. For instance, it has been reported that an increase of PTP1B into the hypothalamus facilitates the storage of body fat [20]. Moreover, neuronal *Ptp1b*^−/−^ (also known as *Ptpn1*^−/−^) mice have reduced weight and adiposity, and increased activity and energy expenditure [21]. Since EVs can pass through the blood–brain barrier, it is possible that they can transmit PTP1B at the central level regulating food intake and energy expenditure such as the hypothalamus. Similarly, it has been shown that the increase in PP2A activity in adipose tissue leads to increased fatty acid synthesis [22]. Further work is necessary to corroborate this hypothesis.

High-level expression of PTP1B accounts for the insulin resistance induced by IR lEVs in adipocytes. However, because IR lEVs inhibit insulin signalling in vivo in mouse liver but not in cultured hepatocytes, several hypotheses can be proposed. First, HepG2 cells are hepatocarcinoma cells and this may contribute to the observed differences. Second, the ability of lEVs from IR participants to decrease insulin sensitivity in hepatocytes may be time-dependent. Indeed, HepG2 cells were treated for 24 h compared with mice that received two EV injections in 14 days, and then insulin was directly injected into the vena cava, exposing hepatocytes rapidly to insulin. Third, lEVs from IR participants might indirectly decrease insulin sensitivity in mouse liver, more specifically in hepatocytes, by activating surrounding microenvironmental cells such as macrophages [23]. Indeed, IR lEVs reduce insulin signalling in hepatocytes, probably via macrophage secretion of soluble factors, by a PTP1B inhibitor-sensitive mechanism. Additionally, IR sEVs affect insulin signalling in both adipocytes and hepatocytes through a common pathway associated with high-level expression of PP2A.

Furthermore, the presence and activity of phosphatases are quite high in n-IR EVs; these EVs had no effect on insulin response. It is possible that the amount of phosphatases in n-IR EVs is not sufficient enough to affect insulin signalling, whereas in IR EVs, the threshold is reached and they induce insulin resistance. We cannot exclude other mechanisms compensating the phosphatase activity in n-IR EVs.

### Limitations

Here, we analysed the effects of EVs from insulin-resistant individuals on insulin resistance in male mice; however, the effects in female mice remain to be determined. Indeed, it has been described that female mice are protected against insulin resistance [24] and it is plausible that the effects of IR EVs are less pronounced in female mice. In addition, women are more sensitive to insulin than men; however, this metabolic advantage gradually disappears after menopause [25]. Although we did not analyse the implications of sex in the present study, it should be noted that the mean age of participants corresponds to the age of menopause in women, suggesting that our findings may be generalisable to both sexes. Further investigations are needed to decipher whether the effects of EVs from insulin-resistant individuals are sex-dependent.

The molecular link between phosphatases transported by EVs and their tropism to specific tissues needs further investigation. Indeed, we have previously shown that IR sEVs did not affect insulin response in endothelial cells [1]. Several approaches may be used but none of them provides optimal results. Although constitutive *Ptp1b*^−/−^ mice are available, the whole-body knockout implies that these mice are protected from insulin resistance. Constitutive deletion of PP2A is embryonically lethal in mice (for review, see [26]). Another option is to use tissue-specific *Ptp1b*- or *Ppp2ca*-knockout mice; however, in the present study, circulating EVs expressing both phosphatases are derived from several cell-type origins. For instance, lEVs from adipocyte-specific PTP1B knockout mice [27] did not rescue palmitic-acid-evoked insulin resistance in 3T3L1 adipocytes (S. Ali, M. Delibégovic, M. Carmen Martínez, unpublished results), suggesting that adipose tissue-derived EVs were not involved in the effects observed in the present study. Nonetheless, we demonstrate for the first time that circulating EVs from insulin-resistant individuals carry active phosphatases responsible for the insulin resistance induced by these EVs. Specifically, we revealed that IR lEVs are enriched in the active form of PTP1B, whereas IR sEVs mainly carried active PP2A. Since the mechanisms regulating lEV and sEV biogenesis are different, it is plausible that they drive the differential encapsulation of each phosphatase in one type of EV depending on their size (lEVs and sEVs). Furthermore, using pharmacological phosphatase inhibitors, we demonstrated the involvement of lEV-carried PTP1B and sEV-carried PP2A from insulin-resistant individuals in the development of insulin resistance. The identification of PTP1B and PP2A as key players in insulin resistance induced by EVs may have potential therapeutic relevance for the maintenance of insulin signalling in individuals with insulin resistance.

## Electronic supplementary material

Below is the link to the electronic supplementary material.

'ESM (PDF 1050 KB)

## Data Availability

All data generated or analysed during this study are available on request from the authors.

## Appendix: Metabol study group

Clinics (Centre Hospitalo-Universitaire d’Angers [CHU], Angers, France)

• Hepatology: Jérôme Boursier, Paul Calès, Frédéric Oberti, Isabelle Fouchard-Hubert, Adrien Lannes

• Diabetology: Séverine Dubois, Ingrid Allix, Pierre-Henri Ducluzeau

• Pneumology: Frédéric Gagnadoux, Wojciech Trzepizur, Nicole Meslier, Pascaline Priou

• Functional vascular explorations: Samir Henni, Georges Leftheriotis, Pierre Abraham

• Radiology: Christophe Aubé

Basic science (SOPAM, U1063, Inserm, UNIV Angers, SFR ICAT, Angers, France)

Ramaroson Andriantsitohaina, M. Carmen Martínez, Soazig Le Lay, Raffaella Soleti, Luisa Vergori

Statistics: Gilles Hunault

Biological resource centre: Odile Blanchet, Belaid Sekour

Coordination: Jean-Marie Chrétien, Sandra Girre

## Abbreviations

EVs: Extracellular vesicles
GSK3β: Glycogen synthase kinase-3β
IR: Insulin-resistant (group)
lEV: Circulating large extracellular vesicle
n-IR: Non-insulin-resistant (group)
PP1: Protein phosphatase 1
PP2A: Protein phosphatase 2
PPP: Platelet-poor plasma
PTP1B: Phosphotyrosine phosphatase 1B
sEV: Circulating small extracellular vesicle
TLR4: Toll like receptor-4
