# Supplementary material for: Circulating extracellular vesicle-carried PTP1B and PP2A phosphatases as regulators of insulin resistance
Source: Diabetologia. 2024 Oct 18;68(1):231–42. doi: 10.1007/s00125-024-06288-0 (PMC11663195; doi:10.1007/s00125-024-06288-0)
Supplement: Supplementary file 1 — 'ESM (PDF 1050 KB) [file 125_2024_6288_MOESM1_ESM.pdf]

Material and Methods

Participants

This study was approved by the Ethics Committee of the University Hospital Centre of Angers (France) (NUMEVOX Cohort, ClinicalTrials.gov registration no. 00997165). Patients at the Department of Endocrinology and Nutrition of the University Hospital of Angers were recruited as participants. After giving informed consent, cohort participants were characterised according to the Homeostasis Model Assessment for IR (HOMA-IR) index. For the present study, participants with pre-existing chronic inflammatory diseases or cancer were excluded. A total of 67 participants were identified as being insulin resistant (IR group, HOMA-IR$\geq$1.7), and 38 participants were identified as non-insulin resistant (n-IR, HOMA-IR<1.7). Baseline characteristics and clinical data for the participants in the n-IR and IR groups are summarised in ESM Table 1. Sex was determined by participant self-report. Race and ethnicity data were not available in this study. Insulin assay was performed with platelet-poor plasma (PPP) of n-IR and IR individuals. Plasma insulin concentration was measured using Elisa Kit (R&D Systems, Oakville, ON) according to the manufacturer's instructions. Absorbance was read at 570 nm with a microplate reader, and insulin concentration (pmol/l) was determined from a standard curve of pure insulin.

EV isolation

Peripheral blood (approximately 20 ml) from n-IR and IR groups was collected in EDTA tubes (Vacutainers; Becton Dickinson, Le Pont de Claix, France) to isolate circulating EVs, as previously described [1,2]. After centrifugation at 260 *g* for 20 min, platelet-rich plasma was separated from whole blood. Subsequently, the platelet-rich plasma was centrifuged at 1500 *g* for 20 min to obtain the PPP. Two hundred µl of the PPP was frozen at -80°C to characterise lEVs by flow cytometry. The remaining PPP was centrifuged at 21,000 *g* for 45 min and the pellet containing lEVs was washed in 0.9% NaCl. lEVs were resuspended in 200 µl NaCl and

stored at 4°C until use. The remaining lEV-depleted PPP was centrifuged at 100,000 *g* for 70 min (Optima MAX-XP ultracentrifuge and MLA-80 rotor, Beckman Coulter, Villepinte, France). The pellet containing sEVs was washed in phosphate-buffered saline (PBS; NaCl 137 mmol/l, KCl 2.7 mmol/l, Na_2HPO_4 10 mmol/l, KH_2PO_4 1.8 mmol/l, pH=7.4). sEVs were resuspended in 200 µl PBS and stored at 4°C until use. All centrifugation steps were performed at 4°C.

Electronic transmission microscopy

EV preparations were first fixed overnight at 4°C with 2.5% glutaraldehyde (LFG Distribution, Lyon, France) in 0.1 M PBS. Then, samples were washed twice in PBS and resuspended in milliQ water. Twenty µl were applied on Formvar®-coated copper grids (Sigma-Aldrich, Saint Quentin-Fallavier. France) for 2 min. EVs were negatively stained with 20 µl uranyl acetate 5% (diluted in ethanol 50%) for 30 s. Briefly, the grids were rinsed with milliQ water and air dried. Grids were then observed with a Jeol JEM 1400 microscope (Tokyo, Japan) at 120 keV.

Characterisation of EV-associated proteins

Purified lEVs or sEVs (10 µg) were separated on 4-15% Criterion TGX precast gels and transferred on nitrocellulose membranes (Bio-Rad Laboratories, Marnes-la-Coquette, France). To characterise EVs, blots were probed with antibodies shown in ESM Table 2.

Flow cytometry

Counting and phenotyping of lEVs were performed by flow cytometry from PPP according to the expression of membrane-specific antigens [2,3]. For this purpose, the following antibodies from Beckman Coulter were used: anti-CD41-PC5 (#6607116) and CD146-PC5 (#A22364). Irrelevant human IgG was used as an isotype-matched negative control for each sample. Ten µl of PPP were incubated with 5 µl of the specific antibodies or the isotype-matched negative controls. Pro-coagulant lEVs exposing phosphatidylserine (5 µl of PPP) were labelled with 2 µl of annexin V-FITC (Miltenyi Biotec, Bergisch Gladbach, Germany, #130-092-052). After

45 min of incubation at room temperature, samples were diluted in 300 µl of sterile 0.9% NaCl or annexin-V labelling buffer (Miltenyi Biotec), and then, flow-count microbeads were added to each sample. Then, samples were analysed by flow cytometry 500 MPL System (Beckman Coulter).

Nanoparticle tracking analysis (NTA)

The size and concentration of sEVs were assessed using the NanoSight NS300 (Malvern Instruments Ltd, Malvern, UK). sEV samples were diluted from 1:100 to 1:500 in a total volume of 1 ml of 0.9% NaCl previously filtered through a 0.22 µm filter. The instrument recorded 5 videos of 60 s per sample in the optimal temperature range from 20°C to 22°C. Data analysis was performed using NTA software 3.1. The sEV concentration was calculated from the total plasma volume for each participant.

Animals

All animal studies were performed using approved institutional protocols (APAFiS n°#22561 and #47728-2024022218479296 v6) and were in accordance with the Guide for the Care and Use of Laboratory Animals, published by US National Institutes of Health (NIH Publication No. 85-23, revised 1996). No animals were excluded from the analyses. Only male animals were used in this study to allow comparability with previous studies and to exclude potential sex- and oestrogen-related effects on insulin resistance. Swiss male mice RjOrl:SWISS (https://janvier-labs.com/en/fiche_produit/swiss_mouse/), 8-10 w-old (Janvier Lab, Le Genest-Saint-Isle, France) were included in this project. All mice were kept in a 12 h light/12 h dark cycle and fed ad libitum with a standard diet. For *in vivo* treatment, mice received an intravenous injection into the tail vein every 7 d during 2 w of: (i) lEVs from n-IR and IR individuals were used at the circulating concentration detected for each individual, as previously described [2-4]. The values of circulating lEVs detected in donors ranged from 1,100 and 134,608/µl of plasma for n-IR participants and 2,397 and 246,055/µl of plasma for IR

participants; (ii) 30 µg sEV proteins per mouse as described by Ying et al. [5]. Then, animals were included in the glucose tolerance test (GTT), insulin tolerance test (ITT), or *in vivo* insulin-stimulated phosphorylation assay. All animals were randomly assigned to groups and all analyses were blinded to numerical coding of samples whenever possible. Animals were weighed before each injection and before their sacrifice. Adipose tissues, including subcutaneous, visceral and epididymal, were harvested and weighed from some animals. Sample size was determined by power analysis. Based on our previous experiments, the sample size was set as n > 4, as mentioned.

Glucose and insulin tolerance tests

The GTT was performed after 12 h of fasting. Fasting glycaemia was measured, then mice received an intraperitoneal injection of 2 g/Kg glucose (Sigma-Aldrich). Glycemia timing evolution was measured until 120 min after glucose injection. For ITT, mice were fasted for 6 h before receiving an intraperitoneal injection of 0.5 IU/kg insulin (Lilly, Neuilly-sur-Seine, France), and blood glucose levels were measured every 15 m for 1 h.

In vivo insulin-induced Akt phosphorylation assay

To evaluate insulin action in tissues, insulin-induced Akt phosphorylation was measured in visceral adipose tissue, and liver as described by Ying et al. [5]. Animals were fasted for 8 h, then anaesthetized with isoflurane (Baxter, Guyancourt, France), and portions of these tissues were harvested to measure basal levels of Akt phosphorylation. Then, mice were injected with 0.75 IU/kg insulin into the vena cava and portions of the liver and visceral adipose tissues were collected at 3 and 7 min, respectively, with these time points predetermined to obtain maximal Akt phosphorylation.

Histological analysis

Adipose tissue and liver were frozen in liquid nitrogen and stored at -80°C until histological analysis. Tissue sections were then stained with haematoxylin-eosin (Sigma-Aldrich).

Quantification of adipocyte size was performed using ImageJ software (https://imagej.nih.gov/ij/, [6]; January 2020).

Cell culture

All cell strains (ATCC, Manassas, VA) were negative for mycoplasma and maintained at 37°C in a humidified incubator with 5% CO_2 for HepG2 and 10% CO_2 for 3T3L1-adipocytes. HepG2 hepatocytes were cultured in DMEM high glucose (25 mmol/l glucose; Sigma-Aldrich) supplemented with 10% FBS (ThermoFisher Scientific, Rockford, IL), 1% penicillin/streptomycin, nonessential amino acids, 2 mmol/l L-glutamine and 0.1 mmol/l sodium pyruvate (all from Lonza, Basel, Switzerland). 3T3L1 pre-adipocytes were differentiated into mature adipocytes 2 d after confluence in DMEM high glucose supplemented with 10% FBS, 1% penicillin/streptomycin, 2 mmol/l L-glutamine and a differentiation cocktail consisting of 1.25 µmol/l dexamethasone, 250 µmol/l 3- isobutyl-methyl-1-xanthine (IBMX) and 250 nmol/l insulin (all for Sigma-Aldrich) for 72 h. Then, cells were cultured with 100 nmol/l insulin until complete differentiation. For the experiments, HepG2, and mature 3T3L1 were serum deprived in DMEM low glucose (5.5 mmol/l glucose; Sigma-Aldrich) supplemented with 0.5% BSA as well as the respective additives described above. Then, cells were treated for 24 h with a circulating concentration of lEVs equivalent to $2.5 x 10^4 \pm 5 x 10^3$ and $4.6 x 10^4 \pm 5.4 x 10^3$ lEVs/µL for n-IR and IR individuals, respectively. For sEV treatment, cells were treated with 10 µg/mL sEVs corresponding to $5.4 x 10^5 \pm 7.9 x 10^4$ and $1.7 x 10^6 \pm 3.1 x 10^5$ sEVs/µL for n-IR and IR individuals, respectively. Palmitic acid (Sigma-Aldrich), a positive control of insulin resistance, was used at 0.3 mmol/l for a final ratio of 4:1. After 24 h of treatment, cells were stimulated with 100 nmol/l insulin for 5 min. Cell-permeable phosphatase inhibitors were used. To block the effect of PTP1B carried by lEVs, two inhibitors of PTP1B acting through different mechanisms at two different concentrations were used to assure that the activity of PTP1B was inhibited [7, 8]. Thus, 50 µmol/l BML-267 (Santa Cruz

Biotechnology, Dallas, TX) or 10 μmol/l MSI-1436 (Toronto Research Chemicals, North York, ON) were added to isolated lEVs for 1 h at 4°C before pelleting the lEVs by centrifugation at 21,000 *g* for 45 min. To inhibit the action of PP2A carried by sEVs, 0.5 nmol/l okadaic acid (Enzo Life Science, Villeurbanne, France) [9] was added to isolated sEVs for 1 h at 4°C. sEVs were pelleted with ExoQuick (System Biosciences, Palo Alto, CA) followed by centrifugation at 1500 *g* for 30 min. Then, cells were incubated with either BML-267-treated lEVs or okadaic acid-treated sEVs for 24 h followed by insulin stimulation with 100 nmol/l insulin for 5 min.

Glycogen assay

After treatment, HepG2 cells were stimulated with 100 nmol/l insulin for 90 min. Then the cells were homogenized in 100 μl ice water and boiled for 5 min to inactivate the enzymes. The glycogen assay was measured using specific kit (Sigma-Aldrich) according to the manufacturer's instructions. Absorbance was read at 570 nm using a microplate reader and glycogen concentration was determined from a standard curve of pure glycogen.

Western blotting

After EV treatment, proteins were separated from lysed cells or tissues (20 μg) on 4-15% Criterion TGX precast gels and transferred to nitrocellulose membranes using Trans-Blot turbo transfer system (Bio-Rad Laboratories). Blots were probed with antibodies against IRS1 (#05-1085) and phospho-IRS1 Tyr 612 (#09-432) (Merck Millipore, Burlington, MA), Akt (#2920) and phospho-Akt Ser 473 (#4060), GSK3β (#9832), phospho-GSK3β Ser 9 (#9323), and GADPH (#sc-32233, Santa Cruz Biotechnology). The membranes were then incubated with the appropriate horseradish peroxidase (HRP)-conjugated secondary antibody (goat anti-guinea pig IgG (H+L), goat anti-rabbit IgG (H+L)-HRP or anti-mouse IgG (H+L)-HRP) (ThermoFisher Scientific or Sigma-Aldrich) diluted in Tris buffer containing 1% Tween-20 and 5% bovine serum albumin.

Phosphatase activity assay

EVs were resuspended in lysis buffer containing 20 mmol/l imidazole, 2 mmol/l EGTA, 1 mmol/l phenylmethylsulfonyl fluoride, and protease inhibitors. Phosphatase activity was measured using a specific pNPP hydrolysis kit (Merck Millipore) according to the manufacturer's instructions (PTP Assay Kit 1, #17-125 for PTP1B and Ser/Thr Phosphatase Assay Kit 1 (K-R-pT-I-R-R), #17-127 for PP2A). Samples were incubated for 10 min before measuring absorbance at 410 nm using a microplate reader.

Human macrophage culture and multiplex immunoassays for macrophage secretome

Human peripheral blood mononuclear cells were isolated by density gradient centrifugation over a Ficoll cushion solution (Histopaque 1077; Sigma-Aldrich) from platelet apheresis of healthy volunteers obtained by the Etablissement Français du Sang (Angers, France) according to a protocol approved by the Etablissement Français du Sang Ethics Committee and in compliance with the Declaration of Helsinki. Written informed consent was obtained from all subjects participating in the study. Monocytes were purified by MACS immunomagnetic positive selection using the human CD14-microbeads isolation kit (Miltenyi Biotec), resulting in preparations with a purity of ≥95%, as determined by flow cytometry after staining with anti-PE/Cy7-CD14 mAb (Biolegend, #BLE325618). To generate macrophages, $1x10^6$ primary monocytes/ml were cultured for 6 d in 6-well culture plates with RPMI 1640 (Sigma-Aldrich) supplemented with 10% EV-depleted FBS (ThermoFisher Scientific), 1% penicillin/streptomycin, nonessential amino acids, 2 mmol/l L-glutamine and 100 ng/ml of the maturation factor, human M-CSF premium grade (Miltenyi Biotec). On day 3 of culture, half of the medium was replaced, and then macrophages were treated with lEVs for 24 h in the absence or presence of the PTP1B inhibitor, MSI-1436 (10 µmol/l). Supernatant fractions were used for analysis of multiple secreted proteins using multiplex assays according to the manufacturer's protocol (Meso Scale Discovery, Rockville, MD).

Quantification of neutral lipids

To measure the amount of intracellular lipids, cells were stained with Oil-red-O (Sigma-Aldrich). Briefly, after 24 h of treatment with lEVs, sEVs, in the absence or in the presence of phosphatase inhibitors or palmitic acid, cells were fixed with 4% paraformaldehyde for 20 min and then incubated with Oil-red-O for 1 h at room temperature. The excess stain was removed by extensive washing and Oil-red-O was extracted from the stained cells using pure isopropanol and absorbance was measured at 510 nm using a microplate reader (PerkinElmer, Waltham, MA). Adipocyte and lipid droplet size were quantified by Image J software [6] (https://imagej.nih.gov/ij/, February 2023).

Quantitative real-time PCR analysis

RNA was extracted from mouse adipose tissue and quantitative real-time PCR (qPCR) was performed using a CFX96™ Real-Time PCR Detection System and SYBR Green detection (Bio-Rad). Target expression was normalized to the endogenous gene and expressed relative to the control by 2−ΔΔCt [ΔCt = Ct (target gene) − Ct (endogenous gene); ΔΔCt = ΔCt for each sample - ΔCt for control], where Ct is the cycle threshold. The following specific primers (Sigma-Aldrich) were used: *Srebp* (forward GGAGCCATGGATTGCACATT, reverse GCTTCCAGAGAGGAGGCCAG), *Fas* (forward TGCTCCCAGCTGCAGGC, reverse GCCCGGTAGCTCTGGGTGTA), *Fabp4* (forward AACACCGAGATTTCCTT, reverse ACACATTCCACCACCAG), *Gadph* (forward CAAGGTCATCCATGACAACTTTG, reverse GGCCATCCACAGTCTTCTGG).

Statistical analysis

Comparisons were made between all conditions for each panel. Statistical analysis was performed using ANOVA, followed by Kruskal Wallis for multiple comparisons of > 2 groups, and when the comparison was between 2 groups, data were analysed using the Mann-Whitney U test. When measurements were repeated in the same group of animals, a two-way ANOVA followed by Tukey's multiple comparison was performed. No experiment-wide multiple test

correction was applied. Values shown in the text and figures represent the mean ± SEM or median (interquartile range) as indicated in the figure legends. Each dot represents an EV isolation from one individual. A p value ≤ 0.05 was considered statistically significant and presented in figures and tables. When $p > 0.05$, p values were not shown. Statistical analysis was performed using GraphPad Prism 8.0 (GraphPad Software Inc., San Diego, CA).

References

1. Ali S, Malloci M, Safiedeen Z, et al (2021) LPS-enriched small extracellular vesicles from metabolic syndrome patients trigger endothelial dysfunction by activation of TLR4. Metabolism 118:154727. https://doi.org/10.1016/j.metabol.2021.154727

2. Perdomo L, Vidal-Gómez X, Soleti R, et al (2020) Large extracellular vesicle-associated Rap1 accumulates in atherosclerotic plaques, correlates with vascular risks and is involved in atherosclerosis. Circ Res 127:747-760. https://doi.org/10.1161/CIRCRESAHA.120.317086

3. Agouni A, Lagrue-Lak-Hal AH, Ducluzeau PH, et al (2008) Endothelial dysfunction caused by circulating microparticles from patients with metabolic syndrome. Am J Pathol 173:1210-1219. https://doi.org/10.2353/ajpath.2008.080228

4. Safiedeen Z, Rodríguez-Gómez I, Vergori L, et al (2017) Temporal cross talk between endoplasmic reticulum and mitochondria regulates oxidative stress and mediates microparticle-induced endothelial dysfunction. Antioxid Redox Signal 26:15-27. https://doi.org/10.1089/ars.2016.6771

5. Ying W, Riopel M, Bandyopadhyay G, et al (2017) Adipose tissue macrophage-derived exosomal miRNAs can modulate in vivo and in vitro insulin sensitivity. Cell 171:372-384. https://doi.org/10.1016/j.cell.2017.08.035

6. Schneider CA, Rasband WS, Eliceiri KW (2012) NIH Image to ImageJ: 25 years of image analysis. Nat Methods 9:671–675. https://doi.org/10.1038/nmeth.2089

7. Lund IK, Hansen JA, Andersen HS, Møller NPH, Billestrup N (2005) Mechanism of protein tyrosine phosphatase 1B-mediated inhibition of leptin signalling. J Mol Endocrinol 34:339-51. https://doi.org/10.1677/jme.1.01694.

8. Lantz KA, Emeigh Hart SG, Planey SL, et al (2010) Inhibition of PTP1B by trodusquemine (MSI-1436) causes fat-specific weight loss in diet-induced obese mice. Obesity (Silver Spring) 18:1516-23. https://doi.org/10.1038/oby.2009.444.

9. Swingle M, Ni L, Honkanen RE (2007) Small-molecule inhibitors of ser/thr protein phosphatases: specificity, use and common forms of abuse. Methods Mol Biol 365:23-38. https://doi.org/10.1385/1-59745-267-X:23.

ESM Table 1: Characteristics of insulin resistant (IR) participants compared to non-IR participants

	**n-IR**	**IR**	**P**
**Number**	38	67	
**Mean age (years)**	56.9 ± 1.3	55.9 ± 1.1	NS
**Sex ratio (male/female)**	30/8	36/31	
**Body mass index (kg/m^2)**	26.3 ± 0.5	32.5 ± 0.5	<0.001
**Waist circumference**	92.3 ± 1.3	111.3 ± 1.4	<0.001
**Systolic blood pressure (mmHg)**	127.5 ± 2.7	134.8 ± 1.5	0.013
**Diastolic blood pressure (mmHg)**	79.4 ± 1.7	79.9 ± 1.1	NS
**Hb1Ac (mmol/mol) [%]**	37.9 ± 0.7 [5.6 ± 0.07]	43.9 ± 1.0 [6.2 ± 0.1]	<0.001
**Blood glucose levels (mmol/l)**	5.2 ± 0.1	6.3 ± 0.15	<0.001
**Blood insulin levels (pmol/l)**	38.4 ± 4.7	112.4 ± 13.5	<0.001
**HOMA-IR**	0.94 ± 0.12	6.3 ± 0.8	<0.001
**HDL (mmol/l)**	1.38 ± 0.05	1.07 ± 0.04	<0.001
**LDL (mmol/l)**	3.2 ± 0.14	2.77 ± 0.12	0.045
**Triglycerides (mmol/l)**	1.11 ± 0.08	2.11 ± 0.15	<0.001
**Plasma lEVs (events/µl plasma)**	$2.9 \times 10^4 \pm 5.2 \times 10^3$	$4.7 \times 10^4 \pm 4.8 \times 10^3$	0.0013
$CD41^+$ lEVs (events/µl plasma)	$2.6 \times 10^4 \pm 4.7 \times 10^3$	$4.3 \times 10^4 \pm 4.6 \times 10^3$	0.0011
$CD146^+$ lEVs (events/µl plasma)	235 ± 91	629 ± 185	NS
**Treatments (%)**			
Oral glucose-lowering agents	5	49.2	
Antihypertensive agents	24	62.3	
Statin	18	42	

Homeostatic model assessment (HOMA) is a used to quantify insulin resistance (HOMA = blood glucose levels x blood insulin levels/22.5). NS = not significant. Values are expressed as mean ± SEM.

ESM Table 2: List of reagents and software used

Antibodies (dilution)	Target	Supplier	Catalog number, RRIDs
1:1000	CD9	Santa Cruz Biotechnology	Cat# sc-13118; RRID:AB_627213
1:1000	CD63	Santa Cruz Biotechnology	Cat# sc-59286, RRID:AB_784278
1:1000	CD81	Santa Cruz Biotechnology	Cat# sc-7637; RRID:AB_627190
1:1000	Grp94	Santa Cruz Biotechnology	Cat#393402; RRID:AB_2892568
1:1000	GM130	Santa Cruz Biotechnology	Cat#55591; RRID:AB_1124984
1:1000	ApoA1	Santa Cruz Biotechnology	Cat#376818; RRID:AB_2797313
1:1000	PTP1B	Generated by M. Delibegovic	N/A
1:1000	PP2A	GeneTex	Cat# GTX133710, RRID:AB_2887069
1:1000	Perilipin	Progen Biotechnik	Cat# GP29, RRID:AB_2892611
1:1000	PP1	Santa Cruz Biotechnology	Cat#7482; RRID:AB_628177
1:5,000	β-actin	Sigma-Aldrich	Cat# A5441; RRID:AB_476744
1:40	CD41-PC5	Beckman Coulter	Cat# 6607116
1:40	CD146-PC5	Beckman Coulter	Cat# A22364
1:1000	phospho-IRS1 Tyr 612	Merck Millipore	Cat# 09-432, RRID:AB_1163457
1:1000	IRS1	Merck Millipore	Cat# 05-1085, RRID:AB_1977296
1:1000	Akt	Cell Signaling	Cat# 2920, RRID:AB_1147620
1:2000	phospho-Akt Ser 473	Cell Signaling	Cat# 4060, RRID:AB_2315049
1:1000	GSK3β	Cell Signaling	Cat# 9832, RRID:AB_10839406
1:1000	phospho-GSK3β Ser 9	Cell Signaling	Cat# 9323, RRID:AB_2115201
1:20	CD14-PC7	Biolegend	Cat# 325618, RRID:AB_830691

1:5,000	β-actin	Sigma-Aldrich	Cat# A5316, RRID:AB_476743
1:1000	GADPH	Santa Cruz Biotechnology	Cat#sc-32233, RRID:AB_627679
1:15,000	Goat anti-Guinea Pig IgG (H+L) Secondary Antibody, HRP	ThermoFisher Scientific	Cat# A18769, RRID:AB_2535546
1:20,000	Goat anti-Rabbit IgG (H+L) Secondary Antibody, HRP	ThermoFisher Scientific	Cat# 31460, RRID:AB_228341
1:15,000	Monkey anti-Mouse IgG (H+L)-Peroxidase	Sigma-Aldrich	Cat# SAB3701105
**Biological samples**			
	Human monocytes	Etablissement Français du Sang	N/A
**Chemicals, peptides, and recombinant proteins**			
	ExoQuick	System Biosciences	Cat# EXOTC50A
	BML-267	Santa Cruz Biotechnology	Cat# sc-205605
	human M-CSF	Miltenyi Biotec	Cat# 130-096-491
	MSI-1436	Toronto Research Chemicals	Cat# T891970
	CD14 beads	Miltenyi Biotec	Cat# 130-050-201, RRID:AB_2665482
	iQ™ SYBR® Green Supermix	Biorad	Cat# 1708880
	Okadaic acid	Enzo Life Sciences	Cat# ALX-350-003
**Critical commercial assays**			
	Human/Canine/Porcine Insulin Quantikine ELISA Kit	R&D Systems	Cat# DINS00
	Glycogen assay kit	Sigma-Aldrich	Cat# MAK016
	PTP Assay Kit 1	Merck Millipore	Cat# 17-125
	Ser/Thr Phosphatase Assay Kit 1	Merck Millipore	Cat#17-127
	Multiplex cytokine assay	Meso Scale Discovery	Cat# K15067L
	Annexin V-FITC kit	Miltenyi Biotec	Cat# 130-092-052

**Experimental models: Cell lines**			
	HepG2	ATCC	Cat# HB-8065, RRID:CVCL_0027
	3T3L1	ATCC	Cat# CL-173; RRID:CVCL_0123
**Experimental models: Organisms/strains**			
	Swiss mice	Janvier Lab	Cat# RjOrl:SWISS
**Oligonucleotides**			
	Srebp (forward GGAGCCATGGATTGCACATT, reverse GCTTCCAGAGAGGAGGCCAG)	Sigma-Aldrich	N/A
	Fas (forward TGCTCCCAGCTGCAGGC, reverse GCCCGGTAGCTCTGGGTGTA)	Sigma-Aldrich	N/A
	Fabp4 (forward AACACCGAGATTTCCTT, reverse ACACATTCCACCACCAG)	Sigma-Aldrich	N/A
	Gadph (forward CAAGGTCATCCATGACAACTTTG, reverse GGCCATCCACAGTCTTCTGG)	Sigma-Aldrich	N/A
**Software and algorithms**			
	ImageJ	Schneider et al., 2012	https://imagej.nih.gov/ij/
	Nanoparticle tracking analysis software 3.1	Malvern Instruments	N/A
	GraphPad Prism 8.0	GraphPad Software Inc	N/A

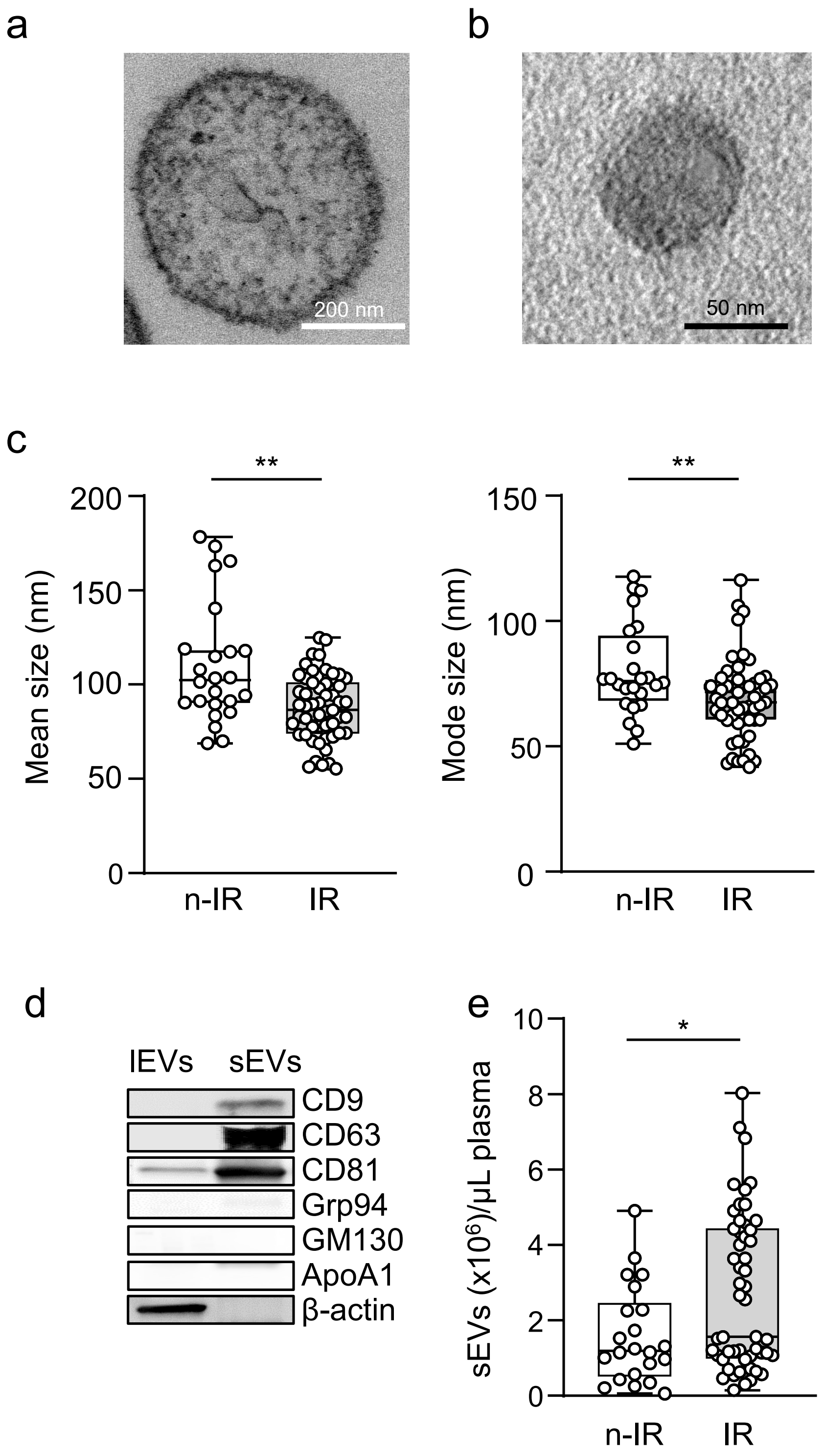

ESM Fig 1: Characterisation of circulating lEVs and sEVs.

(a, b) Transmission electronic microscopy images of lEVs (a) and sEVs (b) from participants; (c) sEV size measured by nanoparticle tracking analysis; (d) Immunoblot representing expression of CD9, CD63, CD81, Grp94, GM130, ApoA1, and β-actin in circulating EVs from participants; (e) Circulating concentration of sEVs from non-insulin resistant (n-IR) and insulin-resistant (IR) individuals. Data are shown as medians and interquartile ranges. Mann-Whitney U test was carried out. **p* <0.05, ***p*<0.01.

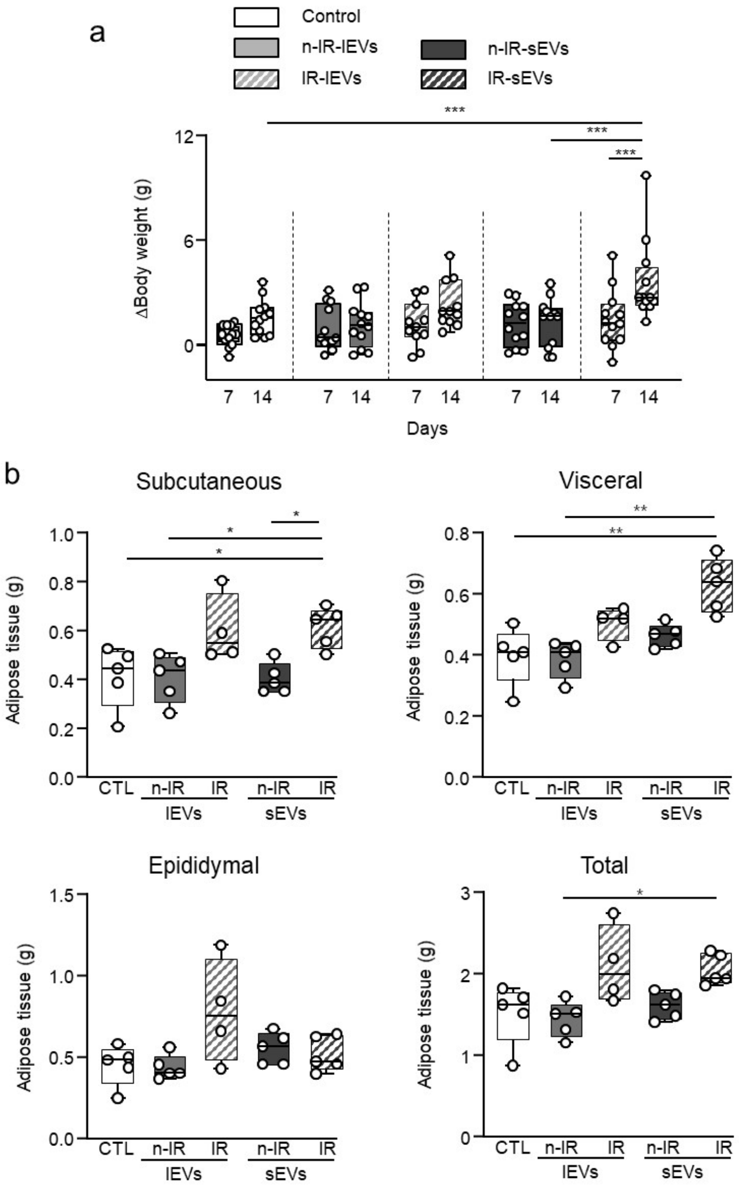

ESM Fig. 2: EVs from individuals with insulin resistance (IR) promote adiposity.
(a) Body weight changes of mice injected with vehicle (control), circulating levels of lEVs or 30 µg sEVs from non-insulin resistant (n-IR) and insulin-resistant (IR) participants. n=11-12 mice/group. Data are shown as medians and interquartile ranges. *p* values were determined by the repeated-measures two-way ANOVA followed by a Tukey's multiple comparisons. (b) Adipose tissue weight at the sacrifice. *p* values were determined by ANOVA followed by Kruskal Wallis test. *$p < 0.05$, **$p < 0.01$, ***$p < 0.001$. CTL, control.

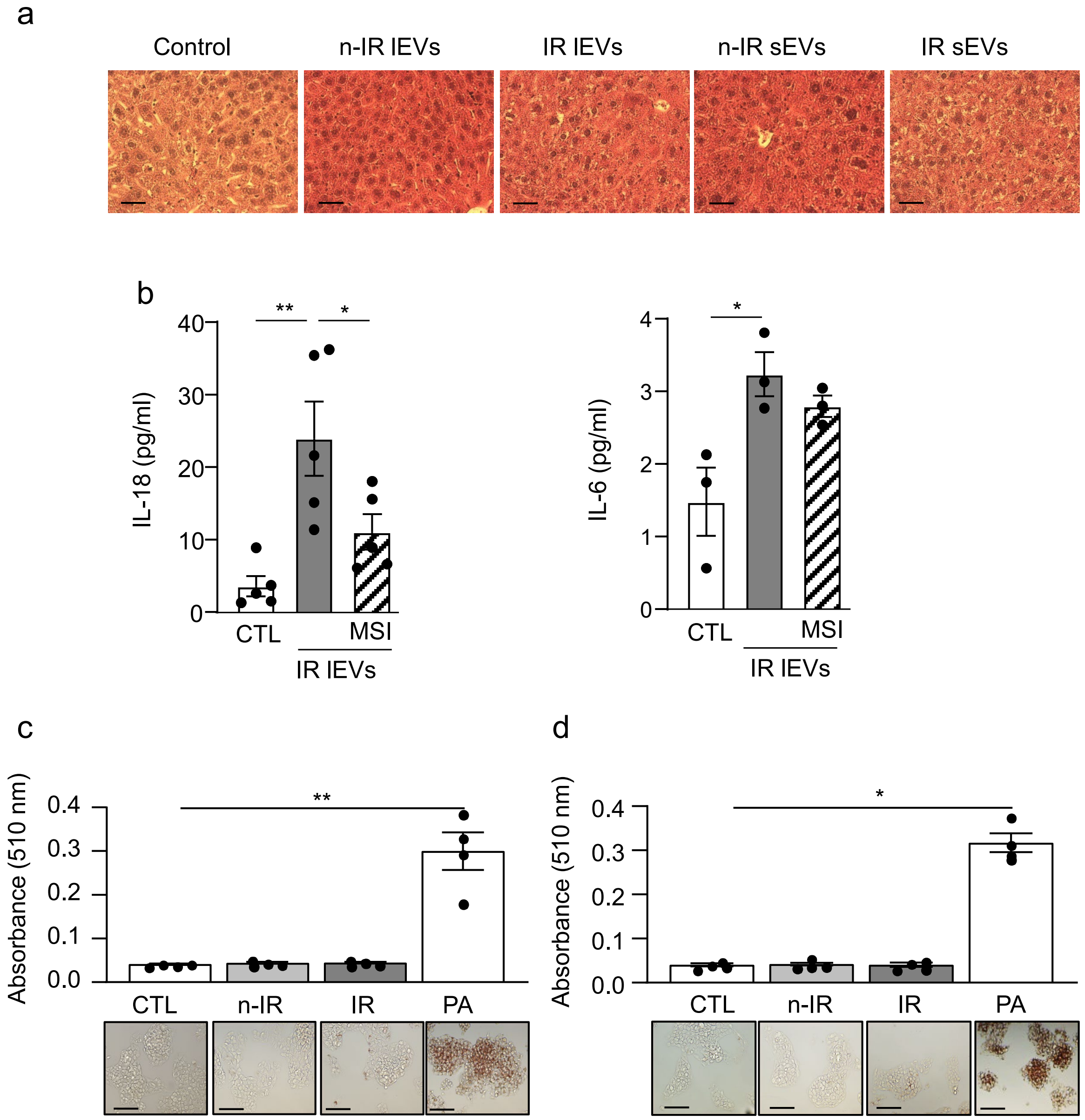

ESM Fig 3: lEVs and sEVs do not modify cell morphology in mouse liver tissue or lipid accumulation in HepG2 cells but increase pro-inflammatory cytokines IL-18 and IL-6 in macrophages.

(a) Mice were injected every 7 days for 2 weeks with either lEVs or sEVs from non-insulin resistant (n-IR) and insulin-resistant (IR) individuals, then liver was collected, and tissue section were stained with haematoxylin-eosin. Scale bar=50 µm; (b) Human macrophages were treated for 24 hours with IR lEVs pre-incubated with 10 µmol/l MSI-1436. n=5 independent experiments; (c, d) Cells were incubated with 300 µmol/l palmitic acid (PA), lEVs (c) or sEVs (d) from n-IR and IR participants. before Oil-red-O staining. n=4 independent experiments. Scale bar=50 µm. Data are shown as mean and SEM. ANOVA was carried out followed by Kruskal Wallis test. *p<0.05; **p<0.01. CTL, control; MSI, MSI-1436; PA, palmitic acid.

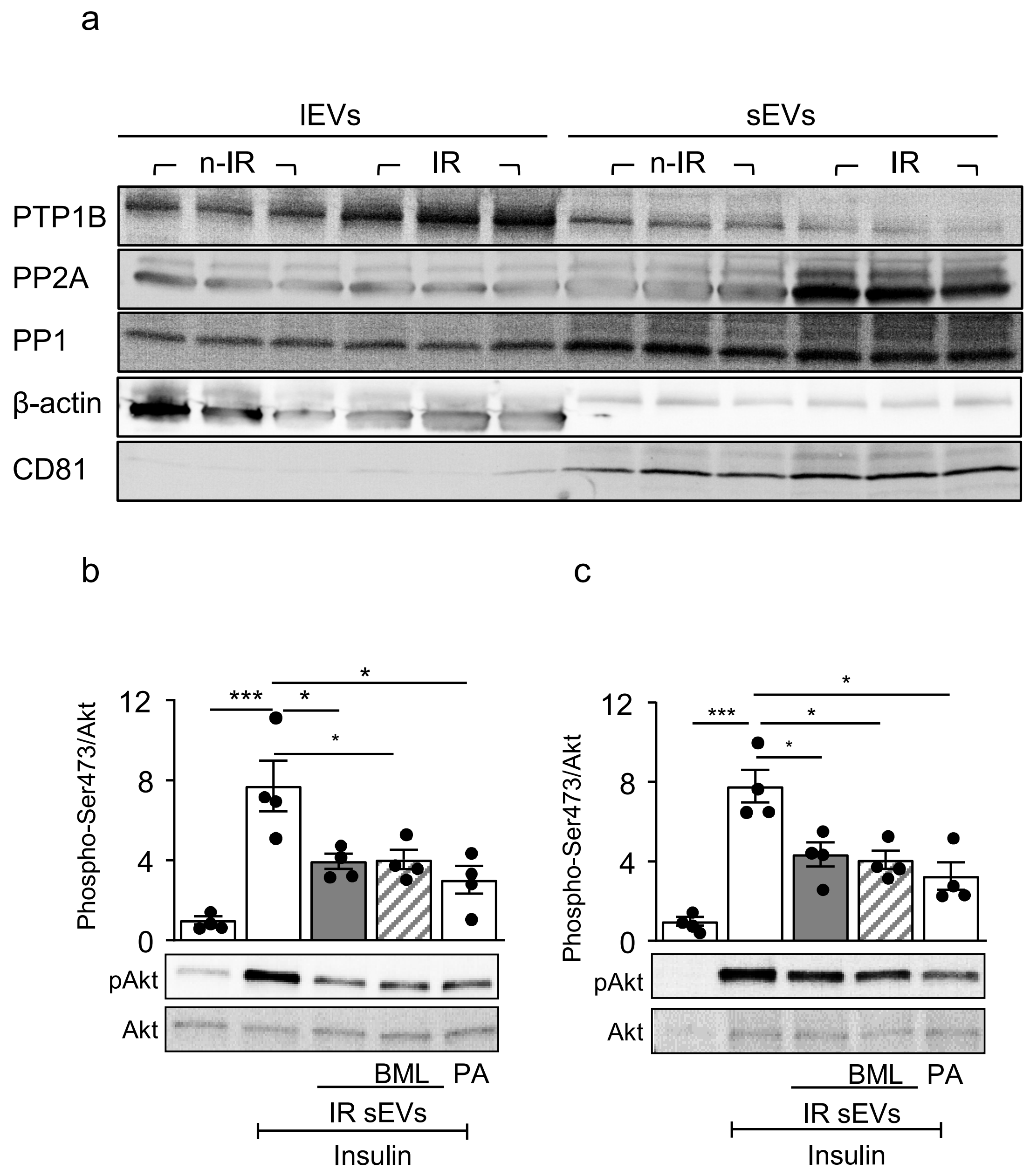

ESM Fig 4: Phosphatase content in circulating lEVs and sEVs. Inhibition of PTP1B has no effect on insulin resistance induced by insulin resistant (IR) sEVs in adipocytes and hepatocytes.

(a) PTP1B, PP2A and PP1 protein expressions in circulating EVs from non-insulin resistant (n-IR) and insulin-resistant (IR) individuals. ß-actin and CD81 were used as control of protein loading in lEVs and sEVs, respectively. n=3 human samples/group. (b, c) Immunoblot and quantification of phosphorylation of Akt Ser473 in 3T3L1-adipocytes (b) and HepG2 (c) treated for 24 hours with IR sEVs pre-incubated with 50 µmol/l BML-267. n=4 independent experiments. Data are shown as mean and SEM. ANOVA was carried out followed by Kruskal Wallis test. *p<0.05 and ***p<0.001. BML, BML-267; PA, palmitic acid; PP1, protein phosphatase 1; PP2A, protein phosphatase 2A; PTP1B, protein tyrosine phosphatase 1B.
